# Kids motor performances datasets

**DOI:** 10.1016/j.dib.2020.106582

**Published:** 2020-11-30

**Authors:** Ahmad Bisyri Husin Musawi Maliki, Mohamad Razali Abdullah, Ahmad Nadzmi, Mohamad Amirur Rafiqi Zainoddin, Intan Meily Puspitasari, Nur Faizatul Amira Jibril, Nur Amirah Nawi, Siti Musliha Mat-Rasid, Rabiu Muazu Musa, Zarizal Suhaili, Noor Aishah Kamarudin, Syed Kamaruzaman Syed Ali

**Affiliations:** a East Coast Environment Research Institute, 21300 University of Sultan Zainal Abidin, Gong Badak Campus, Terengganu, Malaysia; b Faculty of Applied Science Social University of Sultan Zainal Abidin, 21300 University of Sultan Zainal Abidin, Gong Badak Campus, Terengganu, Malaysia; c Faculty of Sport Science, Sultan Idris Education University, 35900, Tanjong Malim, Perak Darul Ridzuan, Malaysia; d Centre for Fundamental and Continuing Education, University Malaysia Terengganu, Terengganu, Malaysia; e Faculty of Applied Science Social University of Sultan Zainal Abidin, 21300 University of Sultan Zainal Abidin, Gong Badak Campus, Terengganu, Malaysia; f Department of Mathematics and Science Education, Faculty of Education, University of Malaya, 50603 Kuala Lumpur, W.Persekutuan Kuala Lumpur, Malaysia

**Keywords:** Motor performance, Biology growth, Physical fitness test, Primary school, Sports talent identification

## Abstract

These datasets described the data of the Motor Performance Index for 7 years old kids in Malaysia based on Malaysia's physical fitness test SEGAK. This database has been designed and created with data analysis to create the index from the factor and variable of the test and the test was conducted in the majority of the national primary school in Malaysia. Gender, state of origin, and residential location of the school were the factors used to categorize the participant of the test. The factor of age, weight, height, body mass index (BMI), power, flexibility, coordination, and speed were used for the measurement to relate with the participant's physical fitness. Kids Motor Performances Index data can be reused for talent identification in sport talent scout and to create a baseline for kid's biology growth specifically in gross motor skills and cognitive growth measurement.

## Specification Table

**Table:** 

Subject	Sport Sciences, Therapy, and Medicine
Specific Area Subject	Sport Science (Talent Identification, Social Functionality, Gross Motor Skills, Biological Growth)
Type of data	Table Graph Chart Excel file
How data was acquired	Data was collected by performing Malaysia's physical fitness test (SEGAK)
Data Format	Mixed (raw and pre-processed)
Parameters for data collection	A total of 1998 from 7 years-old kids who are in national primary regional school and participating in Malaysia's physical fitness test (SEGAK) were selected as the sample for this research.
Description of data collection	This data was collected from physical fitness tests at the national primary regional school and send to the National Sports Institute (ISN), act as a data collection center than in turn, the data sent to the East Coast Environment Research Institute (ESERI) to be analyzed.
Data Source Location	Malaysia
Data Accessibility	Data with the article Repository Name: Mendeley Direct URL to data: https://data.mendeley.com/datasets/ntv67hkp4p/draft?a=6d15a80f-71df-40e9–9a2b-6a83fbf9ea6b
Related research article	A.B.H.M. Maliki, M.R. Abdullah, H. Juahir, F. Abdullah, N.A.S. Abdullah, R.M. Musa, S.M. Mat-Rasid, A. Adnan, N.A. Kosni, W.S.A.W. Muhamad, N.A.M. Nasir, A multilateral modeling of Youth Soccer Performance Index (YSPI), in: IOP Conf. Ser. Mater. Sci. Eng., 2018. https://doi.org/10.1088/1757–899X/342/1/012057.

## Value of Data

• Data herein can be used to identify patterns, trends, and data abnormality for Talent Identification Program purposes which could assist in determining kids motor performances as well as kids physical fitness index.
• The data is especially valuable to the parents, teachers, talent scouters, coaches, trainers, and health educators.
• The relevant stakeholders could reuse the data for benchmarking, policy making, as well as program initiation to cater to kids well-being.

## 1 Data Description

This research was conducted to understand work in various issues, such as motor performance index, kid's segmentation, kid's strength, leg muscle power, kid's flexibility, kid's coordination, and kid's speed. Consequently, these datasets provide useful information based on survey data on the motor activity of kids.

**Fig. 1:**
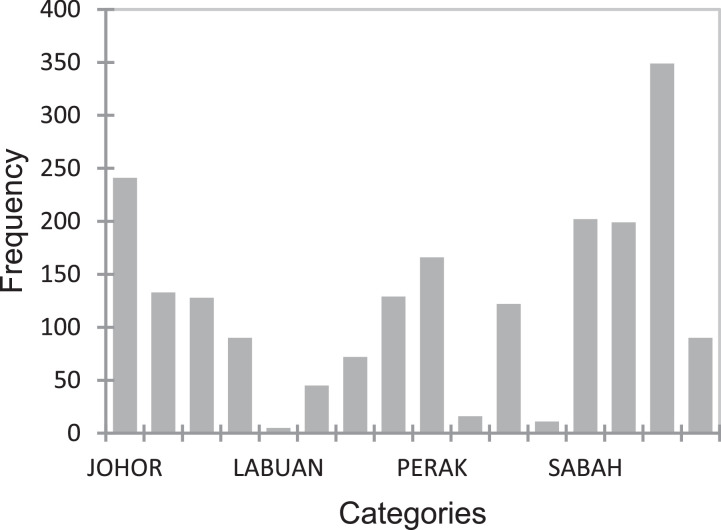
Data state frequency.

**Fig. 2:**
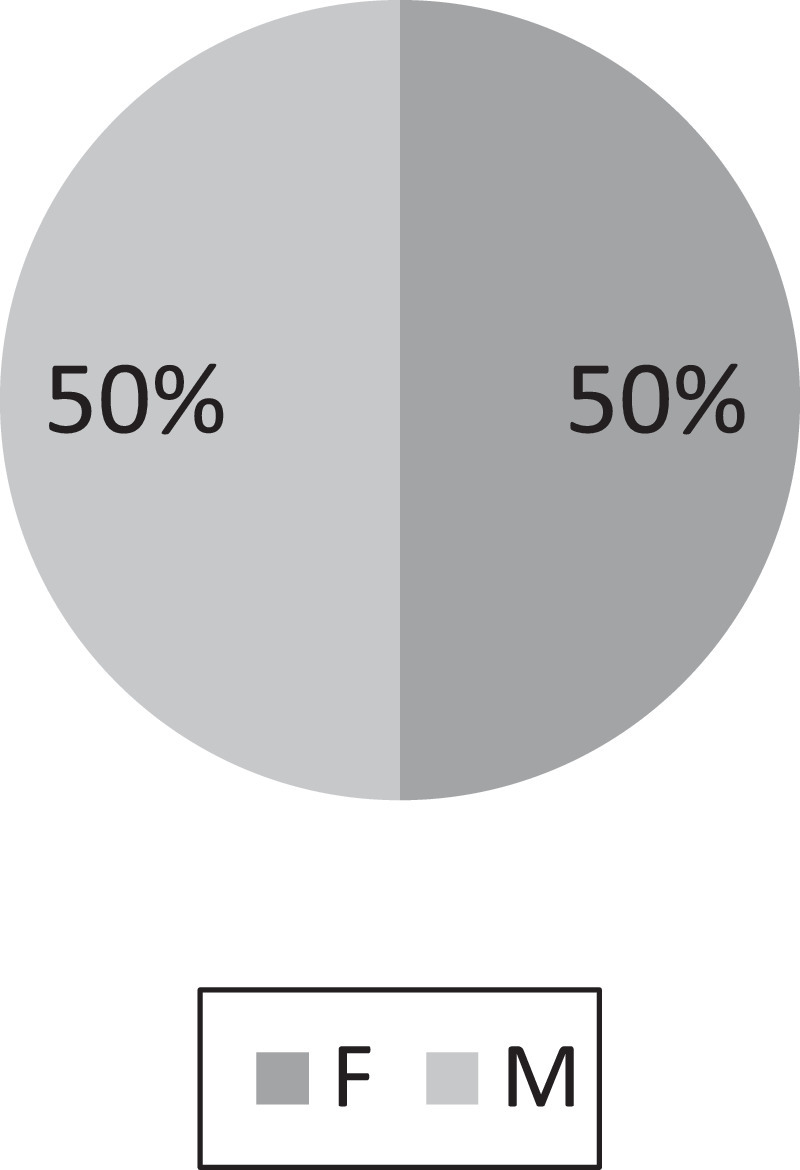
Data gender frequency.

**Fig. 3:**
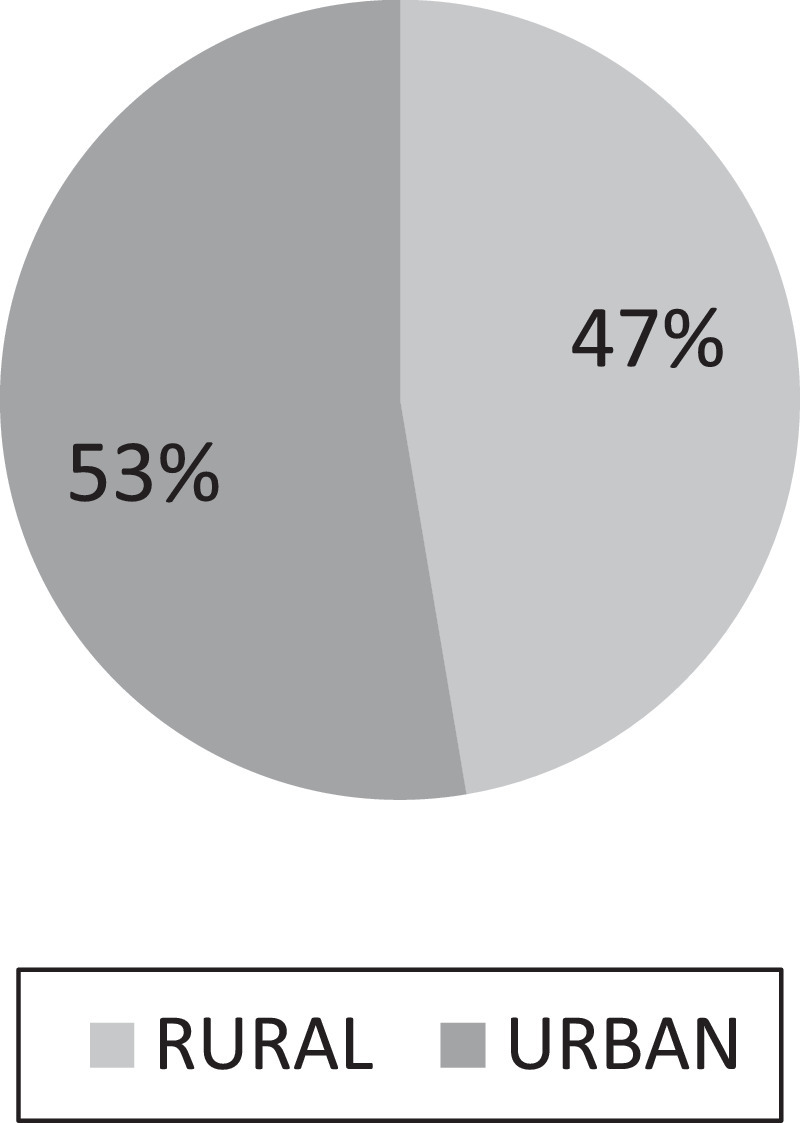
Data residential frequency.

**Fig. 4:**
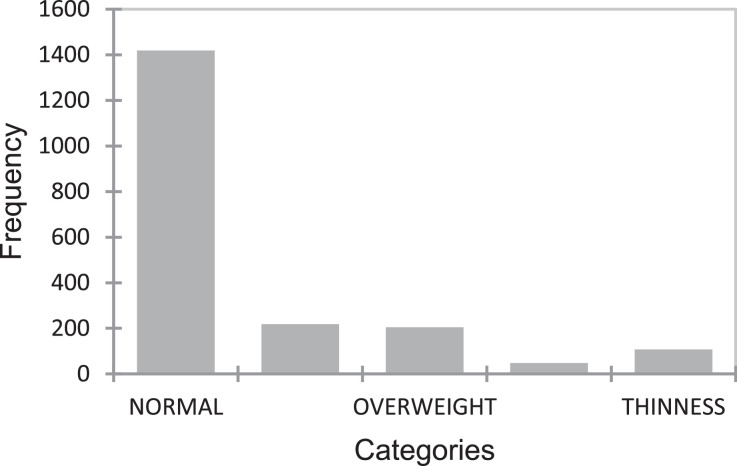
Data class BMI frequency.

Table 1 displays the summary data of the variable used in the physical test for quantitative data, while Figs. 1–4 displays the summary data of qualitative variables. Moreover, the physical test data contains a total of 8 variables namely; age, weight, height, BMI, power, flexibility, coordination, and speed. It is worth to highlight that the data also constitute the various locations of the kids as shown in Fig. 1–4. Raw data are available in the Mendeley dataset.

**Table 1:** Data summary of quantitative data.

Variable	Obs	Mean	Standard deviation (n)	Variance (n)
Age	1998	7.06	0.05	0
Weight (kg)	1998	22.21	5.41	29.28
Height (cm)	1998	118.26	5.97	35.66
BMI (kg/M^2^)	1998	15.77	3.06	9.38
Power (cm)	1998	96.2	17.59	309.4
Flexibility (cm)	1998	26.26	4.93	24.3
Coordination (no.)	1998	4.08	2.78	7.73
Speed (s)	1998	5.16	0.71	0.5

## 2 Experimental Design, Material and Methodology

### 2.1 Participants

The participants who had been involved in this study were 7 years old kids in Malaysia. The participants were identified via cross-sectional collection across all the states in Malaysia. Due to the necessity of physical education and health subjects in the Malaysian school teaching and learning session, participation was required to undergo their SEGAK test. This data was obtained from physical fitness tests conducted by the representative teacher of physical education at the National Regional Primary School and sent to the National Sports Institute (ISN), operating as a data-gathering center, then the data was sent to the East Coast Environment Research Institute (ESERI) to be interpreted by the researchers.

The current data involved a total of 1998 participants (male=999; female=999). Parents, guardians, teachers and administrators, and researchers were granted informed consent to clarify a few items before data collection, such as testing methods, research aims, and others [1]. Participants who took part in the physical fitness tests were eligible for data collection. In the consent form, some of the essential aspects which participants need to fill out such as participant's personal information and contact details (emergency contact information if any emergency problems occur during participating in the test), medical information, and approval of parent's permission. The details were for research purposes only. The researcher has never revealed all the personal details.

### 2.2 Power-standing broad jump (SBJ)

The participants standing behind a line marked in the area. The participants will take a two-foot take-off jump and land with the two-foot-take-off, with the arms swinging back and the legs bent rhythmically at around 90° forward [2]. The participants must jump as far as possible without falling backward and must landing with both feet. This test will not be considered if the participant fails to follow the procedure [3]. Three trials were allowed and the furthest score was recorded.

### 2.3 Speed-twenty-meter speed test (20MR)

The participants sprint through a specific distance with the recorded time taken. The range from the starting line to the finishing line was 20 m [4]. The counting and completion of the period started when each foot passed the starting point when either foot approached the end point [5]. Participants must ensure a fixed stance before starting to run, starting from a fixed position, with the foot back to the starting line while without making any motions. The duration during the participant's run from the starting line to the finish line was recorded by the conductor using a stopwatch.

### 2.4 Flexibility-sit and reach (SAR)

The participants must sit on the ground with both perfect straight leg posture, while their knees on the ground and their feet facing against the flat surface of the sit and reach box [6]. The participant's sides of the knees are held straight against the floor with minimum force by the conductor. With the participant's hands-on top of each other with palms face down, the participants slowly pushing forward their fingertips while moving the measuring slide as far as possible across the measuring line. The reach must be sustained for a minimum of two seconds while the conductor recording the distance [7]. The conductor must make sure participants are not making any jerky movements and the fingertips and legs are in a fixed straight position. Test results shall be taken in multiples of 0.5 cm.

### 2.5 Coordination-hand wall toss (HWT)

Participants have to stand straight behind the line marked and facing the target face with a range of 1 m against the target face at the wall. The gap from the wall to the target face is 1 m down [8]. The ball is tossed with one hand by moving the underarm towards the wall and attempting to catch the ball with the other hand. The ball is first thrown to the wall and caught back by the dominant hand, then catch back by the non-dominant hand. After that, the participants throw back the ball with a non-dominant hand and catch back by dominant hands and continues until the trial is used up [9]. The participant is given for 10 times trial. The number of throws that were caught will be recorded.

## Credit Author Statement

**Ahmad Bisyri Husin Musawi Maliki**: Methodology, Data curation, Resources, Validation, Investigation, Funding acquisition, Visualization, Writing review & editing. **Mohamad Razali Abdullah**: Validation, Investigation, Visualization, writing original draft, Writing review & editing. **Ahmad Nadzmi**: Conceptualization, Methodology, Resources, Validation, Investigation, Supervision, Project administration, Writing review & editing. **Mohamad Amirur Rafiqi Zainoddin**: Investigation, Data curation, Writing review & editing. **Intan Meily Puspitasari**: Investigation, Data curation, Writing review & editing. **Nur Faizatul Amira Jibril**: Validation, Supervision, Project administration, Writing review & editing. **Nur Amirah Nawi**: Investigation, Data curation, Writing review & editing. **Siti Musliha Mat-Rasid**: Conceptualization, Methodology, Resources, Validation, Investigation, Supervision, Project administration, Funding acquisition, Visualization, Writing review & editing. **Rabiu Muazu Musa**: Investigation, Supervision, Project administration, Funding acquisition, Visualization, Writing review & editing. **Zarizal Suhaili**: Supervision, Project administration, Funding acquisition**. Noor Aishah Kamarudin**: Funding acquisition. **Syed Kamaruzaman Syed Ali**: Funding acquisition.

## Ethics Statement

Informed written consent from participants was obtained by the writers. Participants were mandatory due to the requirement of physical education subject in Malaysia's school teaching and learning session. The authors value human subjects' privacy rights as an ethical research team. Therefore, the data submitted does not classify participants and has been entirely anonymous and contains no data to identify participants.

## Declaration of Competing Interest

The authors confirm that they have no established conflicting financial interests or personal relationships that have influenced the research reported in this article, or may be perceived to have influenced it.

## Appendix Supplementary materials

Image, application 1
